# Institutional Notes and News

**Published:** 1909-10-02

**Authors:** 


### Institutional Notes and News.

### VIENNA JUBILEE HOSPITAL.

The new city hospital of Vienna, built in commemoration of the Emperor's Jubilee, will cost more than half a million sterling, and promises to be the most magnificent healing institution in Europe. For financial reasons the more costly pavilion style has been abandoned, and instead the hospital will be built around an immense court, thus avoiding the old style of close, compact building. The new hospital, the plans for which have just been completed, will have the most modern scientific and technical equipment, and form a model institution in every way. It will stand in park-like grounds of considerable extent, on which £10,000 is to be spent in the preliminary laying out. There will be accommodation for 1,000 patients, and provision will be made for extending the buildings whenever necessary.

### GREAT NORTHERN HOSPITAL.

The Great Northern Hospital appeals for £12,000. The hospital costs £17,000 a year, while the income from investments and annual subscriptions is no more than £3,000. Last year there was a deficiency of £4,600, and this year one of £3,000 is anticipated. The Committee owes the hospital bankers £11,800. Unless sufficient money can be obtained to pay for current needs at least fifty beds will have to be closed by the end of the year. Commenting on the appeal, the local newspaper remarks: "Great as would be the pity, it would be better to take that course than to increase the debt. The appeal is made at an unfortunate time. It is only to be expected that everybody who has any money will hold on to it as long as there is doubt as to what the Government will allow him to do with it and how much of it they intend to seize."

### THE LATE MR. W. F. M'MULLEN.

At a special meeting of the South Charitable Infirmary and County Hospital, the Chairman, the Rt. Rev. Dr. Meade, Bishop of Cork, moved a vote of sympathy to the family of the late Mr. W. F. M'Mullen, who, up to the time of his demise, was a member of the Committee of Management, and on a great many public boards in Cork. Of all the institutions he was connected with, Mr. M'Mullen's pet one was the South Infirmary. The name of M'Mullen had been associated with the South Infirmary for nearly half a century. His father worked on the board as trustee and a member of the House Committee for 32 years. The resolution read as follows: "We have learned with profound regret of the death of our esteemed colleague and friend, Mr. William F. M'Mullen, and desire to record our sense of his great loss to this institution, and to the City of Cork. Mr. M'Mullen, who has passed away at a time of life when his usefulness was a most valuable asset, was an ardent worker, and constant attendant, not alone on this board, but at the weekly meetings of the House Commiteee, having been elected on the Joint Committee of Management in the place of his honoured father, Mr. J. W. M'Mullen, who had been a close friend of the South Infirmary and trustee for 32 years. Mr. William F. M'Mullen's work during the last seven years for this hospital was marked by unfailing attention and devotion to its best interests, and he brought to bear a wise and mature judgment in the deliberations of the management. We desire to convey to his son, Mr. W. J. M'Mullen, to his brother, Mr. Alfred M'Mullen, and the other members of the family, the assurance of our deepest sympathy in their great sorrow."

### EXTENSIONS AT THE GLASGOW CANCER HOSPITAL.

The large extensions to the Glasgow Cancer Hospital, which were opened last week, will form a valuable addition to this institution, and supply what has long been felt to be a necessity. The site of the buildings in Garnethill, on one of the highest parts of the city, is an excellent one. Planned throughout on the lines of a modern hospital, the buildings, when completed, will comprise all the departments required for the treatment of cancer. Each room receives the maximum of light and air, and a flat roof has been provided where patients may exercise and be as much as possible in the open air. Fine views of the hills are obtained, and on a clear day Ben Lomond is easily seen. There are four large wards, each containing eight beds. A convalescent ward is also provided, and nine single wards, which will be used for patients requiring quiet and isolation. An average space of 1,750 cubic feet per patient is given in all wards. The whole of the top flat of the north-east portion of the building has been arranged as a research department. Facilities for the scientific investigation of cancer have been provided in the new wing, and the research department, with its chemical laboratory and other rooms, will be found to contain all the needed accommodation. The architects for the work were Messrs. James M. Monro and Son, Mr. John Dixon being consulting engineer, and Mr. William Muir clerk of works.

### COSSHAM HOSPITAL.

According to the Bristol *Mercury*, the medical staff has met the full committee of this Hospital in consultation in order to decide upon the date of the re-opening of the institution. The views of the medical staff were placed at considerable length before the committee. The staff agreed to render in future their services to the Hospital without any monetary honorarium whatever, and it was decided that the Hospital should be re-opened almost immediately, though the definite date was not decided upon.

The institution will, however, be re-opened on the same lines as when it was temporarily closed last March; that is with the two ground floor wards open to admit patients, so that beds will be provided for about twenty patients altogether. The committee fully realise that their income of £2,600 per annum, which will be the actual amount when the capital has been re-invested, will have to support double this number of patients before the charges of undue costliness can be effectively cleared. It is declared that the actual cost of the building and upkeep has been practically double that of any other institution of the same size in England. The entire cost of the building alone was £35,321, a figure which does not compare very favourably with that of other hospitals, such as Blackpool Hospital, 50 beds, £25,000; Bradford (Yorks), 50 beds, £18,000; Darlington, 55 beds, £23,000; Bournemouth, 52 beds, £18,000, giving an average cost of £23,143, which includes in each case the cost of the building of an out-patients' department, which does not exist at the Cossham Hospital. When the hospital was being equipped, the medical staff was asked to make suggestions as to what was necessary, and were led to understand that money was plentiful. The members of the medical staff say that after 1908, when they were first aware of the financial condition, they suggested to the managing body that it would be an assistance to the committee if one of their staff was made a member of that committee, having the same power as any other member. That was often done in other hospitals, and they claim it as not an unreasonable offer to make considering that they were giving their professional services free. It is claimed that the committee will need the co-operation of the medical staff to effect such economies as will enable the larger scheme of the hospital to be carried out as soon as possible.

### NEW INFIRMARY AT HOMERTON.

On Saturday last the new infirmary and workhouse provided by the City of London Guardians was formally opened at Homerton, N.E. The new buildings are the outcome of a scheme which has been before the City Guardians for some time for reducing their Poor-law expenditure by giving up their present institution at Bow and concentrating all their work at Homerton. The practical result of this scheme is that the Guardians find themselves with a workhouse and infirmary sufficient for all their purposes, designed and equipped for 681 indoor inmates and 63 officers. The cost has been between £50,000 and £60,000, while £3,500 has been expended on the provision of a lunatic block. The institution is equipped with all the latest appliances. At the opening ceremony Mr. J. B. Wild (Chairman of the Amalgamation Committee) presided, and said that the new infirmary had been built at a cost far below that of any similar existing building with equal equipment. The cost of the new buildings and contingent works came out at the extremely low figure of about £154 per bed. The Rev. T. Greear, before declaring the buildings open, said that in 1869, owing to the changing conditions of life in the City of London, the three unions then existing—the East, the West, and the City—were united, and their work concentrated at Homerton and Bow. In the 40 intervening years the marvellous expansion of the business and commerce of the City had practically ousted its resident population, and consequently greatly diminished the area for which Poor-law provision had to be made. Those vast economic and social changes gradually made themselves felt in the steady decrease in the number of inmates both at Homerton and Bow. They had now successfully concentrated their energies in Homerton, which would be sufficient for all their work. This development would lead to considerable economy, the saving being estimated at £6,000 to £7,000 a year. The architect (Mr. Pridmore) then presented a gold key to the Chairman of the board, with which he unlocked the door of the administrative block, and, after entering and declaring the buildings open, unveiled a commemorative tablet.

### COWES COTTAGE HOSPITAL.

Princess Henry of Battenberg, as President of the Frank James Memorial Cottage Hospital, East Cowes, presided at the annual meeting of that institution. The annual report expressed gratification that the extended accommodation afforded by the new ward for women, the provision of which was suggested by the President, had greatly increased the usefulness of the institution. The committee also placed on record their respectful and grateful thanks to Princess Henry for her continued interest in and generous support of the hospital. A pleasing feature of the year was that some £300 had been contributed to the funds of the hospital by the workmen of Messrs. J. S. White and Co., Ltd. Mr. Shedden, the Treasurer, proposed a hearty vote of thanks to Princess Henry for her unceasing and kindly interest in the good work and continued prosperity of the hospital. He said that ever since the establishment of that institution her Royal Highness had not only manifested the most kindly interest in the patients and the nursing arrangements, but had actively associated herself with the management of the hospital by presiding at their annual and committee meetings. The thanks of the meeting were also accorded to Mr. Leigh Densham for assisting the funds of the hospital by throwing open the grounds of Norris Castle to the public to view the Naval Review, £55 being thus realised from fees for admission.

### WORKMEN'S COMPENSATION.

Sir Thomas Oliver, F.R.C.P., LL.D., Professor of Physiology in the University of Durham, delivered the opening address of the post-graduate course, promoted by Glasgow Royal Infirmary, Mr. J. D. Hedderwick, LL.D., chairman of the board of managers, presiding. In the course of his address Professor Oliver said that the sphere of practical medicine had become widened by the action of the State. The rise of the factory system, the nation's care for the commonweal, and the medical inspection of school children, had opened up careers for members of their profession and brought medicine into touch with social problems in a manner previously undreamed of. One unsatisfactory aspect of our educational system was seen in the absence of any attempt to deal personally with the physical and mental disabilities of the pupils. The amount of educational waste shown from statistics was astounding, and showed how faulty our educational system had been. By the passing of the Workmen's Compensation Act human life had by enactment of Parliament a money value assigned to it in the case of accident. It was an unfortunate circumstance that the Workmen's Compensation Act had brought into evidence a class of lawyer and medical practitioner whose behaviour was neither creditable to themselves nor to the professions to which they belonged. Unless a doctor was thoroughly convinced of the reality of an illness and the genuine basis of a claim for compensation under the Act he should not advise the case to be taken into court. If there was one thing in the modern movement which required to be carefully watched it was that medicine should not cease to be a profession and become a trade. In labour matters trade unionism was credited with having improved the conditions of labour and of having bettered the lot of the working classes, but the rigid system as opposed to piece-work with uniform wages for all its members was undoubtedly the weak point of the system. It tied the hands of the best workers. Trade unionism was a levelling down; it was opposed to all that was progressive and was contrary to evolution. Much as they objected to place the unfit workman on the same footing as the fit workman, strange to say the object of medicine and surgery was frequently to act in opposition to natural selection and the law of the survival of the fittest.

### SHEFFIELD ROYAL INFIRMARY.

The extensions which are being carried out at the Sheffield Royal Infirmary are progressing rapidly, the building of the new block having already reached the top of the windows of the second storey. Unfortunately, however, the funds are not coming in as rapidly as the work of extension is being promoted. Approximately, at least £40,000 will be needed to pay for the new buildings, but the sum received or promised to date amounts to only £31,000. The accommodation at the Infirmary is totally inadequate to the demands made on the institution. At the present time there are 256 patients in the institution, a number in excess of the accommodation actually provided, while last week only 37 out of 51 applications for beds could be entertained on that account.

### PLAGUE OBSERVATION HOSPITAL.

An observation hospital has been erected off the Grand Junction Road, near Port Adelaide. It hase been built of galvanised iron and wood, with double walls, lined on the inside with small fluted iron. It stands 18 inches above the crown of the road, and some low-lying ground on its western side is being filled in. The building contains one large ward that can be subdivided, a smaller ward, and nurses' room, and separate from these, but under the same roof, are the kitchen and cook's room, a bathroom, and cellar. Arrangements have been made for the provision of permanent tent structures to be erected within the hospital grounds, so that in case of necessity 60 to 80 beds can be set up at short notice. The hospital is now complete, and is being furnished for the reception of patients.

